# Stem anteversion is not affected by proximal femur geometry in robotic-assisted total hip arthroplasty

**DOI:** 10.1186/s42836-024-00248-0

**Published:** 2024-06-02

**Authors:** Andrea Marcovigi, Gianluca Grandi, Luca Bianchi, Francesco Zambianchi, Marco Pavesi, Fabio Catani

**Affiliations:** 1 Department of Orthopaedic Surgery, Azienda Ospedaliero-Universitaria Policlinico di Modena, University of Modena and Reggio-Emilia, Via del Pozzo 71, Modena, MO 41125 Italy; 2 Ab Medica S.p.A., Cerro Maggiore, MI 20023 Italy

**Keywords:** Total hip arthroplasty, Combined anteversion, Stem anteversion, Robotic arm-assisted surgery

## Abstract

**Background:**

In the present study, the surgeon aimed to align the stem at 5° to 25° in anteversion. The robotic technology was used to measure stem anteversion with respect to proximal femur anteversion at different levels down the femur.

**Methods:**

A total of 102 consecutive patients underwent robotic-arm-assisted total hip arthroplasty (RTHA). 3D CT-based preoperative planning was performed to determine femoral neck version (FNV), posterior cortex anteversion (PCA), anterior cortex anteversion (ACA), and femoral metaphyseal axis anteversion (MAA) at 3 different levels: D (10 mm above lesser trochanter), E (the midpoint of the planned neck resection line) and F (head-neck junction). The robotic system was used to define and measure stem anteversion during surgery.

**Results:**

Mean FNV was 6.6° (SD: 8.8°) and the mean MAA was consistently significantly higher than FNV, growing progressively from proximal to distal. Mean SV was 16.4° (SD: 4.7°). There was no statistically significant difference (*P* = 0.16) between SV and MAA at the most distal measured level. In 96.1% cases, the stem was positioned inside the 5°–25° anteversion range.

**Conclusions:**

Femoral anteversion progressively increased from neck to proximal metaphysis. Aligning the stem close to femoral anteversion 10 mm above the lesser trochanter often led to the desired component anteversion.

## Background

In total hip arthroplasty (THA), stem and cup anteversion are major factors affecting joint range of motion and risk of impingement [1]. Several studies have investigated optimal combined version to avoid impingement and to reduce the risk of hip dislocation: Dorr et al. [2] defined combined anteversion as the sum of stem and cup anteversion, identifying the safe zone as the interval between 25°–45° (+ 5° in female patients), while Widmer and Zurfluh [3] recommended an ideal combined anteversion expressed by the equation “Cup anteversion + 0.7 × Stem anteversion = 37.3°”. Recommendations on stem position and orientation are still debated [2, 4]. For cementless stems, some have advocated that the component version should be dictated exclusively by native femoral anatomy, to obtain the best fit and to reduce stress shielding [5]. This theory is also supported by other authors, who have indicated that the stem version with respect to native version has a minimal influence on postoperative limb axial rotation, and does not affect clinical results [6, 7]. On the other hand, there is a large variability of native femoral neck version, so aligning the stem to native version may result in reduced or excessive combined anteversion and therefore lead to a higher risk of dislocation or impingement [8–11].

An additional variable influencing stem version is the metaphyseal bone version. Several reports have identified a strict relationship between stem anteversion and metaphyseal anteversion, especially when using cementless double-wedge stems [12]. While stem and femoral bone version have been investigated both preoperatively and postoperatively [12–14], no study has assessed the relationship between metaphyseal femoral anteversion and intraoperative stem orientation.

The ability to perform this measurement is now possible with robotic-assisted surgery, which allows for highly accurate measurement of intraoperative stem version in real time [15, 16]. In the present prospective study, preoperative femoral neck and metaphyseal version were calculated on 3D computed tomography (CT) scans and stem anteversion was intraoperatively measured using the robotic-arm-assisted system.

The purpose of this work was twofold. First, it was to assess the relationship between femoral neck anteversion and proximal femur metaphysis anteversion as measured on preoperative CT scans. The second goal was to define the influence of metaphyseal anteversion on stem positioning using a single-wedge uncemented stem during robotic-arm-assisted THA. The hypothesis was that metaphyseal anteversion would increase progressively from femoral neck to the lesser trochanter and that this change in femoral metaphyseal anteversion would facilitate femoral stem anteversion in respect to femoral neck anteversion.

## Methods

A total of 110 consecutive patients undergoing robotic-arm-assisted total hip arthroplasty (RTHA) between February 2019 and September 2020 were enrolled from a single orthopedic center. All patients were diagnosed with end-stage hip osteoarthritis. Patients were excluded if they were diagnosed as having congenital hip dislocation (CHD) (Crowe classification ≥ 1) or having femoral head avascular necrosis which resulted in an unrecognizable hip center of rotation. Five patients were excluded with a diagnosis of severe avascular necrosis and 2 presented a CHD with a Crowe classification of respectively grade 2 and 3. Upon exclusion against the criteria, 1 of the remaining patients declined to sign the informed consent, leaving 102 candidate patients for initial enrollment.

Only candidates to RTHA were considered for the initial enrollment, which excluded patients with history of proximal femur trauma, receiving previous proximal femur surgery and diagnosed with severe osteoporosis, for the unavailability of a suitable stem.

All subjects received CT scans for preoperative case planning with the Mako System (Mako Surgical Corp., Stryker, Fort Lauderdale, FL, USA). Preoperative CT scans of the lower extremities met the following requirements: slices spacing 0.5–1 mm for the pelvis and femur, slice spacing 2.0–5.0 mm for the knee, kV 120–140, and mA 200–250. For all patients, RTHA used the Mako robotic system for implantation of both femoral and acetabular components. A single senior surgeon performed all procedures using a posterior lateral approach. A straight, single-wedge, uncemented stem (Accolade^®^ II, Stryker, Mahwah, NJ, USA) was used in all cases [17].

Pre-defined landmarks were identified by a team of independent technicians, then reviewed preoperatively by the surgeon and used to determine the femoral neck axis (FNA), defined as the line passing from the head center to the neck center and the surgical trans-epicondylar axis (sTEA). The method used to determine FNA was similar to the one described by Reikeras et al. [18]. The femoral neck version (FNV) was then automatically calculated by the robotic system software as the angle formed by FNA and sTEA when these two axes are projected on a plane perpendicular to the anatomic axis of the femur (defined by a proximal canal point and the center of the epicondyles).

**Fig. 1:**
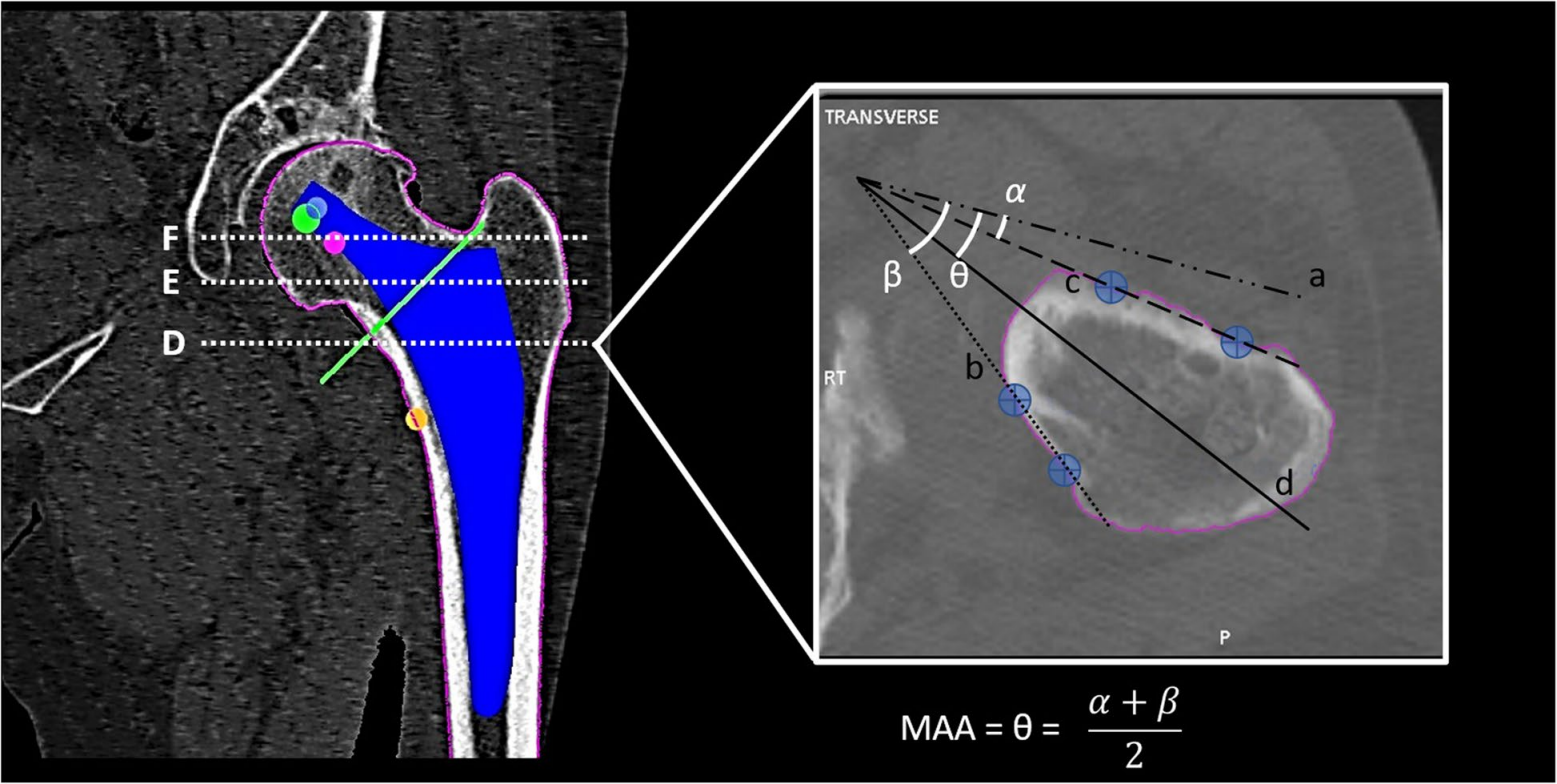
Metaphyseal anteversion measurement at D level: “a” is a line parallel to transepicondylar axis, passing through the point of intersection of “b” and “c” lines; “b” is the line passing through two different points on femoral posterior cortex and represents the posterior cortex axis; “c” is the line passing through two different points on femoral anterior cortex and represents the anterior cortex axis; “d” line is the bisector of the angle formed by anterior and posterior cortex axes and represents the metaphyseal axis. The α angle is equivalent to the angle formed by the anterior cortex axis and the transepicondylar axis, which is defined as anterior cortex anteversion (ACA). The β angle is equivalent to the angle formed by the posterior cortex axis and the transepicondylar axis, and is defined as posterior cortex anteversion (PCA). The θ angle is equivalent to the angle formed between the metaphyseal axis and the transepicondylar axis (metaphyseal axis anteversion: MAA) and, knowing the value of ACA and PCA, could be calculated with the simplified formula shown in the figure

The robotic system software was then used preoperatively to measure femoral metaphyseal anteversion at 3 different levels [12]: D (10 mm above lesser trochanter), E (midpoint of the planned neck resection line) and F (head-neck junction). This involved two steps. First, both posterior cortex anteversion (PCA) and anterior cortex anteversion (ACA) were measured using the method described by Suh et al. [14]. Next, the metaphyseal axis was found as the bisector of the angle subtended by anterior and posterior cortical lines. Metaphyseal axis anteversion (MAA) is the angle formed by the metaphyseal axis and sTEA and was calculated as the mean of ACA and PCA (Fig. 1). All measurements were performed by a single experienced operator using the Mako planning software.

Screw fixation was used for the placement of the femoral and pelvic trackers and morphing acquisitions of the femur and acetabulum were performed to couple CT 3D models and patient’s anatomy. The reported nominal accuracy of the system is 2 mm and 3° for stem measurements, and 2 mm and 5° for robotic cup placement. The integrated optical guide navigation system attached to the prosthesis was used to measure intraoperative stem version (SV) and cup anteversion (CV).

A femur first technique, employing the “Enhanced” Mako robotic software protocol, was used in all cases. This procedure enables the surgeon to acquire the spatial coordinates of femoral bone to perform a direct measurement of femoral anteversion, global offset, and hip length.

A standard 3D planning using Mako System software was performed, setting the stem anteversion at 15° and cup position at 20° of anteversion and 40° of inclination, with slight modifications to avoid excessive acetabular uncoverage.

During the procedure, to achieve an adequate press-fit fixation, the surgeon aimed to achieve an SV between 5° and 25° of anteversion, with the optimal version of 15° as reported by Tönnis et al. [19]. Mako navigation system was employed during femoral preparation to repeatedly measure broach version during progressive size increase.

Cup anteversion target was 15° ± 10° [20], but the cup target was subordinated to the goal of achieving a combined anteversion (the sum of stem anteversion and cup anteversion) between 25°–45° (+5° in female patients) [2].

The study was performed in accordance with the ethical standards of the 1964 Helsinki Declaration and its later amendments and was approved by the local Institutional Review Board (IRB: 70/2018/OSS/AOUMO).

### Statistical analysis

The quantitative variables were expressed as the mean ± standard deviation (SD), minimum and maximum values, whereas the qualitative variables were presented as absolute and percentage frequencies. The relationship of proximal femur anteversion with stem version was assessed by using Pearson’s linear correlation coefficient (σ) and linear regression models. The results of these linear models were reported as regression coefficients (β), while the models’ overall goodness-of-fit was measured as the R-squared index. Finally, odds ratios (OR) from logistic regression models were used to assess the relationship between proximal femur anteversion and the probability of having SV between 10° and 20°.

To validate the tests, a power analysis was performed on linear regression models. For each model, the Z test power was assessed to verify the significance of the coefficient (β) related to the variable included in the model as a predictor. The power was evaluated at a significance level α of 0.05 and was greater than 0.9 for all models. Statistical analyses were performed with R 3.6.3 software (The R Foundation for Statistical Computing, Vienna, Austria) and using a significance level equal to *P* < 0.05.

## Results

A total of 102 patients were enrolled in the study, including 54 females and 48 males, with a mean age of 72.4 years (SD: 9.0, Min: 48, Max: 89) at surgery. Mean femoral neck version was 6.6° (SD: 8.8°, Range: − 28° retroversion to 25° anteversion). Patient age, gender, femoral neck version and final components anteversion are all listed in Table 1.

**Table 1:** Cohort demographics, femoral neck version (FNV) and component anteversion

	**Mean**	**Std. Dev**	**Median**	**Range (Min; Max)**
Age (years)	72.4	9.0	73	48; 89
Females/Males	54/48	-	-	-
FNV (°)	6.6	8.8	7.0	-28; 25
Stem Anteversion (°)	16.4	4.7	16.0	3; 33
Cup Anteversion (°)	24.4	2.3	25.0	18; 31
Combined Anteversion (°)	40.8	3.6	41.0	31; 54

**Fig. 2:**
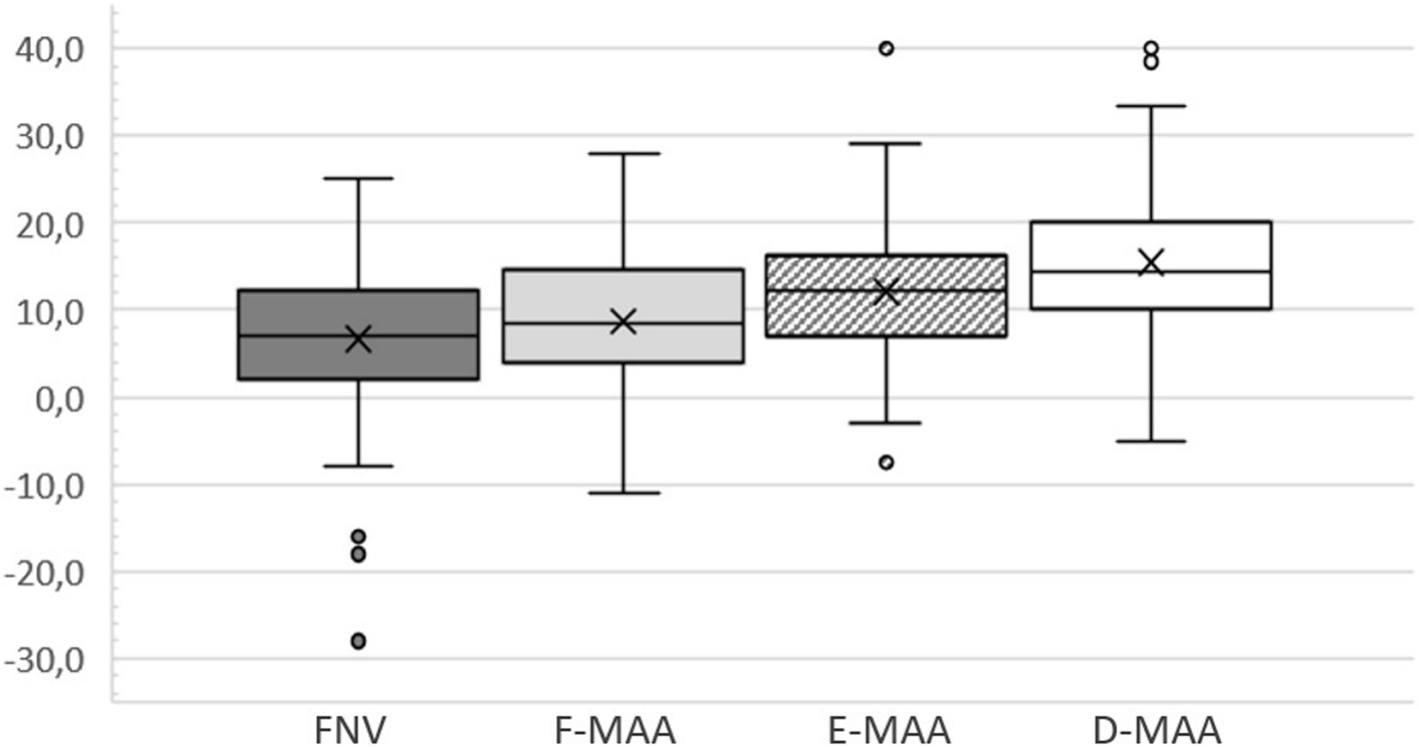
Mean FNV compared with mean metaphyseal axis at F, E and D levels. Femoral anteversion characterized by a progressive increase from proximal to distal metaphysis

The mean MAA was consistently significantly higher than FNV (*P* < 0.05 for F-MAA, *P* < 0.001 for E-MAA and D-MAA) and progressively increased from Level F to Level D (see Table 2 and Fig. 2). Mean MAA was significantly correlated with FNV, with a strong relationship at level F (σ: 0.70, *P* < 0.001) and a moderate relationship at levels E (σ: 0.65, *P* < 0.001) and D (σ: 0.57, *P* < 0.001).

**Table 2:** Proximal femur anteversion at different levels and differences in respect to stem anteversion (SV). The difference between mean SV (16.4° ± 4.7°) and mean MAA at level D was not statistically significant

	**Mean ± SD (°)**	**Difference VS Stem Version ± SD (°)**	***P***
FNV	6.6 ± 8.8	−9.8 ± 8.2	**< 0.001**
F-MAA	8.7 ± 7.9	−7.6 ± 6.7	**< 0.001**
E-MAA	12.1 ± 7.7	−4.3 ± 6.7	**< 0.001**
D-MAA	15.5 ± 7.9	−0.9 ± 7.2	0.16

**Fig. 3:**
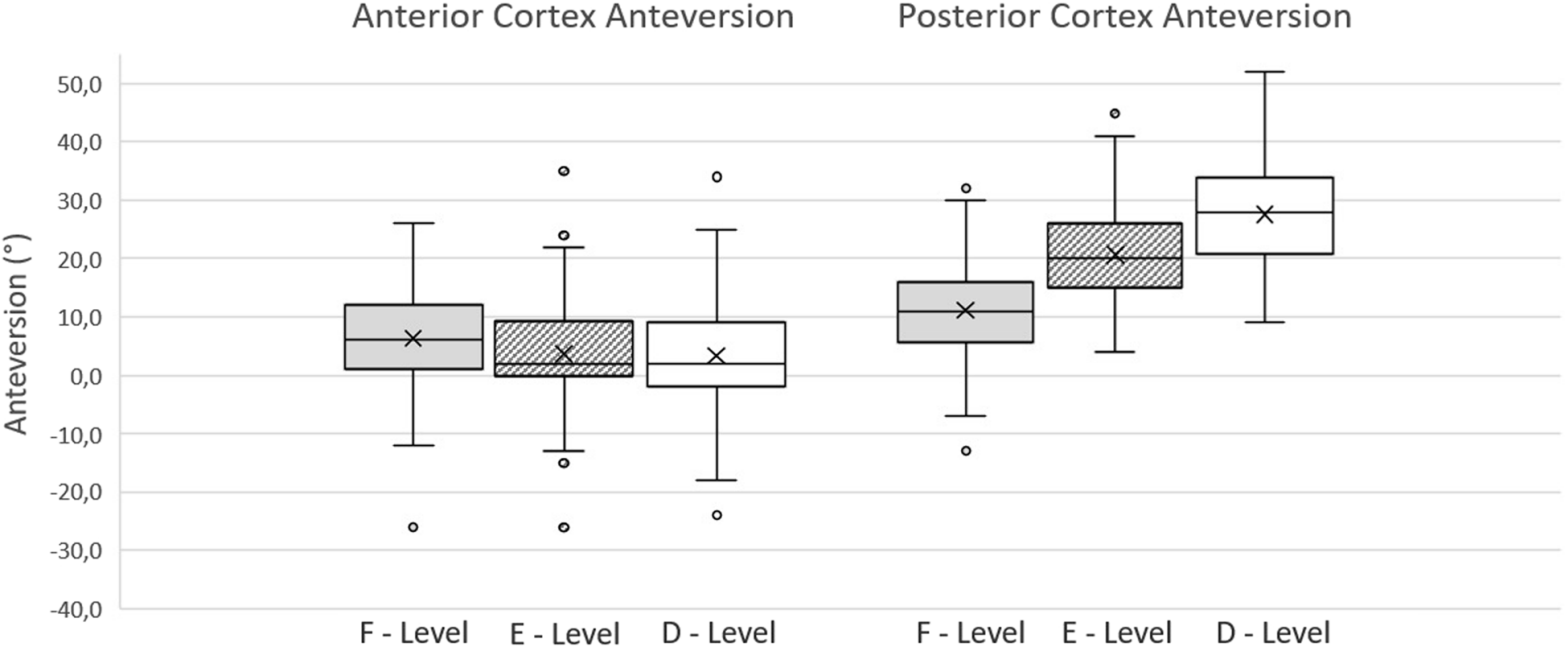
Anterior and posterior cortex mean values. Anterior cortex version was characterized by a slight decrease from level F to level E, while there was no statistically significant difference between E level and D level. Posterior cortex showed a progressive increase in anteversion from level F to level D

Along the 3 levels, PCA markedly increased from level F to level D, with each difference being statistically significant (*P* < 0.001). Conversely, ACA had a statistically significant (*P* < 0.05) decrease from level F to level E with continued decreases from level E to level D that were not statistically significant (*P* = 0.8) (Fig. 3). The overall decrease in ACA from level F to level D was statistically significant (*P* < 0.05).

During surgery, mean final SV was 16.4° (SD: 4.7°, range: 3°–33° anteversion). No statistically significant difference (*P* = 0.16) was found between SV and MAA at Level D, while difference was statistically significant (*P* < 0.001) at Levels E and F, with the SV being consistently higher than MAA (Table 2).

**Fig. 4:**
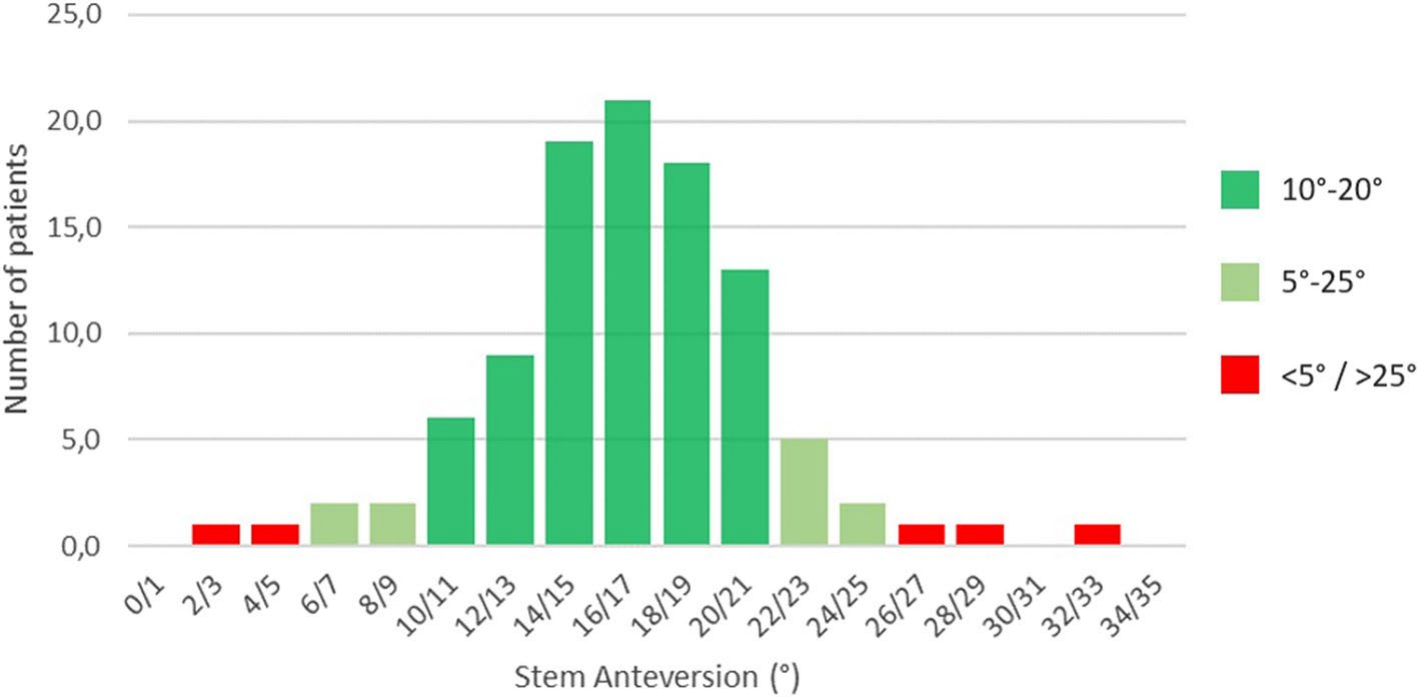
Stem anteversion distribution in the considered population

In 98 cases (96.1%), the stem was positioned within the 5°–25° anteversion range, and in 78 cases (76.5%) the stem was positioned within the 10°–20° anteversion range (Fig. 4). The target range of CV was achieved in 101 cases (99%). The only case outside the target range had a CV of 54°, with a reported SV of 33°. Stem version demonstrated a statistically significant correlation with all measured anatomical parameters of the proximal femur (Table 3). A moderate correlation was found between SV and F and E level MAA, as well as between SV and F and E level ACA. It was not possible to perform Logistic regression for “SV within the range of 5°–25°” due to the paucity of results outside this range. It was instead performed for “SV within the range of 10°–20°” for all anatomical parameters of the proximal femur: none were predictive of “in range” stem version (Table 3).

**Table 3:** Analysis of proximal femur anatomic values in respect to stem anteversion: Stem anteversion relationship was statistically significant in respect to all proximal femur anteversion parameters. In the logistic regression no parameter was significantly associated with stem positioning within the 10°–20° of anteversion target (*OR* Odds Ratio)

				**Relationship of proximal femur anteversion with stem version**					**Probability of stem version 10°***–***20°**	
	**Mean (°)**	**SD**	**Min; Max**	**Linear Correlation**		**Linear Regression**			**Logistic regression**	
				**σ**	***P***	**β**	***R***^**2**^	***P***	**OR**	***P***
FNV	6.6	8.8	−28; 25	0.39	**< 0.001**	0.21	15.6%	**< 0.001**	0.99	0.63
F-PCA	11.2	8.6	−13; 32	0.37	**< 0.001**	0.20	14.0%	**< 0.001**	1.02	0.47
F-ACA	6.4	8.8	−26; 26	0.56	**< 0.001**	0.30	31.8%	**< 0.001**	0.96	0.16
F-MAA	8.7	7.9	−11; 28	0.52	**< 0.001**	0.31	27.1%	**< 0.001**	0.99	0.70
E-PCA	20.7	8.4	4; 45	0.35	**< 0.001**	0.19	12.0%	**< 0.001**	1.00	0.92
E-ACA	3.7	9.4	−26; 35	0.52	**< 0.001**	0.26	26.9%	**< 0.001**	0.97	0.24
E-MAA	12.1	7.7	−7.5; 40	0.51	**< 0.001**	0.31	25.8%	**< 0.001**	0.98	0.47
D-PCA	27.6	8.5	9; 52	0.35	**< 0.001**	0.19	12.5%	**< 0.001**	0.99	0.71
D-ACA	3.4	8.9	−24; 34	0.46	**< 0.001**	0.24	20.8%	**< 0.001**	0.98	0.44
D-MAA	15.5	7.9	−5; 40	0.44	**< 0.001**	0.26	19.7%	**< 0.001**	0.98	0.54

## Discussion

The present study confirmed a strong relationship between femoral neck version (FNV) and the metaphysis axis anteversion (MAA) of the proximal femur. As hypothesized by the author, MAA increased progressively from the femoral neck to the lesser trochanter. Further, as previously identified by Yu et al. [12], we found that the increase in anteversion was due primarily to the increase in posterior cortex anteversion (PCA). Anterior cortex anteversion (ACA) showed only slight level changes, being constant in the neck resection area, and it did not contribute substantially to the overall change in MAA.

The low variability of ACA, regardless of neck resection height, potentially makes it a reliable landmark for traditional THA, when a preoperative CT planning system is available.

Mini-invasive approaches, such as direct anterior approach, often rely on metaphyseal anatomy (especially on posterior cortex orientation) for stem rotational positioning. In these cases 3D planning, procedures proved to reduce complications and could also be used to identify anterior cortex orientation to be used as an intraoperative landmark [21, 22].

Using a combined anteversion technique may help reduce the dislocation rate in THA [23, 24]. Proximal femur anteversion is often ignored in the combined version techniques, because stem positioning is felt to be strictly dictated by proximal femoral anatomy, with the additional assumption that tapered stems have only up to 5° of freedom of rotation and that metaphyseal-filling stems are inflexible with respect to version choice [2, 25, 26].

Hirata et al. [13] demonstrated that, in a straight metaphyseal fitting stem, stem version closely approximates canal version at the lesser trochanter, but they noted that other stem designs could interact differently with the anatomical geometry and fit of the proximal femur.

The present results were obtained with the use of robotic instrumentation, as the surgeon’s estimation of stem anteversion has been demonstrated to have poor precision [27].

Robotic technology demonstrated a high potential to capture data starting from the CT and to the planning and final execution of implant positioning. This technology has been extensively used to assess hip and knee biomechanics [28–30].

In the present study, stem anteversion was not only estimated during preoperative planning but also measured during broaching of the proximal femur to achieve a target of 5°–25° with the optimal value of 15°. This permitted the surgeon to attain a mean stem anteversion of 16.4°, with 96.1% of femoral component aligned within the defined range. One reason the 5°–25° stem anteversion range was adopted, was because low postoperative stem anteversion may increase torsional moment on the prostheses leading to early loosening. Low stem anteversion could also negatively influence combined version, resulting in a higher risk of dislocation or impingement [31].

Widmer and Zurfluh [3], by using a mathematical model, showed that the safe zone of cup placement varies, depending on stem version, highlighting the importance of stem anteversion. A compensatory increase of stem anteversion is also advocated by Incavo et al. [32], to avoid impingement and acetabular uncoverage in patients with retroverted acetabulum.

Stem orientation is usually visually estimated using leg position to approximate the inter-condylar plane position [33]. In the present study, where CT-based planning was performed, it was found that the anterior cortex version remained constant regardless of resection level. Therefore, it can be considered a useful landmark for intraoperative stem rotational alignment, even without using robotic or navigation technology.

Even though the mean stem anteversion was significantly correlated with metaphyseal axis at level D, logistic regression analysis demonstrated that the odds ratio (OR) for obtaining an “in range” stem anteversion was not influenced by any anatomic parameter (metaphyseal axis, anterior cortex, posterior cortex) at any level. It can be assumed that the main reason was that the stem in use, and the intraoperative knowledge of component anteversion, allowed the surgeon to position the stem to his preference, not necessarily following native femoral anteversion.

The present study has several limitations. First, all measurements were taken using robotic instrumentation. This could lead to differences with other studies that are based on postoperative CT scans. Another limitation is that all measurements were taken by a single operator in a single set of records, so it was not possible to assess inter- or intra-observer reliability. Moreover, the present study did not involve clinical data, patients’ outcomes, or implant survivorship. This choice was intended to emphasize intraoperative findings and to avoid confounding data. Lastly, there is no literature evidence or validated technique in support of stem positioning in a defined anteversion using cementless designs, even if many studies support a certain degree of anteversion as an advantage for implant success.

## Conclusion

Femoral anteversion progressively increases from neck to proximal metaphysis, while metaphyseal anterior cortex is constant in the neck resection area, making it a reliable landmark to estimate stem anteversion during surgery. The use of a single-wedge straight stem enabled the surgeon to attain the desired femoral component anteversion with only a slight influence by proximal femur anatomy, even with uncemented fixation. Aligning the stem close to femoral anteversion at 10 mm to a lesser trochanter often leads to satisfactory results in terms of component anteversion, but intraoperative knowledge of stem anteversion is fundamental to achieving the desired stem anteversion and therefore combined anteversion target.

## Data Availability

The datasets used and/or analyzed during the current study are available from the corresponding author on reasonable request.

## References

[1] Harrison CL, Thomson AI, Cutts S, Rowe PJ, Riches PE (2014). Research synthesis of recommended acetabular cup orientations for total hip arthroplasty. J Arthroplasty.

[2] Dorr LD, Malik A, Dastane M, Wan Z (2009). Combined anteversion technique for total hip arthroplasty. Clin Orthop Relat Res.

[3] Widmer KH, Zurfluh B (2004). Compliant positioning of total hip components for optimal range of motion. J Orthop Res.

[4] Yoshimine F (2006). The safe-zones for combined cup and neck anteversions that fulfill the essential range of motion and their optimum combination in total hip replacements. J Biomech.

[5] Hayashi S, Hashimoto S, Matsumoto T, Takayama K, Nishida K, Ishida K, Kuroda R (2017). Stem anteversion mismatch to the anatomical anteversion causes loss of periprosthetic bone density after THA. J Orthop Surg.

[6] Müller M, Abdel MP, Wassilew GI, Duda G, Perka C (2015). Do post-operative changes of neck–shaft angle and femoral component anteversion have an effect on clinical outcome following uncemented total hip arthroplasty?. Bone Joint J.

[7] Uemura K, Takao M, Otake Y, Koyama K, Yokota F, Hamada H (2018). Can anatomic measurements of stem anteversion angle be considered as the functional anteversion angle?. J Arthroplasty.

[8] Shoji T, Yasunaga Y, Yamasaki T, Izumi S, Hachisuka S, Ochi M (2015). Low femoral antetorsion and total hip arthroplasty: a risk factor. Int Orthop.

[9] Sugano N, Noble PC, Kamaric E (1998). A comparison of alternative methods of measuring femoral anteversion. J Comput Assist Tomogr.

[10] Koerner JD, Patel NM, Yoon RS, Sirkin MS, Reilly MC, Liporace FA (2013). Femoral version of the general population: does “normal” vary by gender or ethnicity?. J Orthop Trauma.

[11] Patel AB, Wagle RR, Usrey MM, Thompson MT, Incavo SJ, Noble PC (2010). Guidelines for implant placement to minimize impingement during activities of daily living after total hip arthroplasty. J Arthroplasty.

[12] Yu D, Zeng Y, Li H, Zhu Z, Liu F, Mao Y (2020). Prediction of postoperative stem anteversion in Crowe type II/III developmental dysplasia of the hip on preoperative two-dimensional computed tomography. J Arthroplasty.

[13] Hirata M, Nakashima Y, Itokawa T, Ohishi M, Sato T, Akiyama M (2014). Influencing factors for the increased stem version compared to the native femur in cementless total hip arthroplasty. Int Orthop.

[14] Suh KT, Kang JH, Roh HL, Moon KP, Kim HJ (2006). True femoral anteversion during primary total hip arthroplasty: use of postoperative computed tomography–based sections. J Arthroplasty.

[15] Domb BG, Redmond JM, Louis SS, Alden KJ, Daley RJ, LaReau JM (2015). Accuracy of component positioning in 1980 total hip arthroplasties: a comparative analysis by surgical technique and mode of guidance. J Arthroplasty.

[16] Kouyoumdjian P, Mansour J, Assi C, Caton J, Lustig S, Coulomb R (2020). Current concepts in robotic total hip arthroplasty. SICOT J.

[17] Khanuja HS, Vakil JJ, Goddard MS, Mont MA (2011). Cementless femoral fixation in total hip arthroplasty. J Bone Joint Surg.

[18] Reikeråls O, Bjerkreim I, Kolbenstvedt A (1983). Anteversion of the acetabulum and femoral neck in normals and in patients with osteoarthritis of the hip. Acta Orthop Scand.

[19] van Erp JH, Snijders TE, Weinans H, Castelein RM, Schlösser TP, de Gast A (2022). The role of the femoral component orientation on dislocations in THA: a systematic review. Arch Orthop Trauma Surg.

[20] Lewinnek GE, Lewis JL, Tarr R, Compere CL, Zimmerman JR (1978). Dislocations after total hip-replacement arthroplasties. J Bone Joint Surg.

[21] Post ZD, Orozco F, Diaz-Ledezma C, Hozack WJ, Ong A (2014). Direct anterior approach for total hip arthroplasty: indications, technique, and results. J Am Acad Orthop Surg.

[22] Sariali E, Catonne Y, Pascal-Moussellard H (2017). Three-dimensional planning-guided total hip arthroplasty through a minimally invasive direct anterior approach. Clinical outcomes at five years’ follow-up. Int Orthop.

[23] Nakashima Y, Hirata M, Akiyama M, Itokawa T, Yamamoto T, Motomura G (2014). Combined anteversion technique reduced the dislocation in cementless total hip arthroplasty. Int Orthop.

[24] Widmer KH (2007). Containment versus impingement: finding a compromise for cup placement in total hip arthroplasty. Int Orthop.

[25] Zhang J, Wang L, Mao Y, Li H, Ding H, Zhu Z (2014). The use of combined anteversion in total hip arthroplasty for patients with developmental dysplasia of the hip. J Arthroplasty.

[26] Masumoto Y, Fukunishi S, Fukui T, Yoshiya S, Nishio S, Fujihara Y (2020). New combined anteversion technique in hybrid THA: cup-first procedure with CT-based navigation. Eur J Orthop Surg Traumatol.

[27] Dorr LD, Wan Z, Malik A, Zhu J, Dastane M, Deshmane P (2009). A comparison of surgeon estimation and computed tomographic measurement of femoral component anteversion in cementless total hip arthroplasty. J Bone Joint Surg.

[28] Foissey C, Batailler C, Coulomb R, Giebaly DE, Coulin B, Lustig S, Kouyoumdjian P (2023). Image-based robotic-assisted total hip arthroplasty through direct anterior approach allows a better orientation of the acetabular cup and a better restitution of the centre of rotation than a conventional procedure. Int Orthop.

[29] Kayani B, Konan S, Ahmed SS, Chang JS, Ayuob A, Haddad FS (2020). The effect of anterior cruciate ligament resection on knee biomechanics: changes in flexion-extension gaps, mediolateral laxity, and maximum knee extension. Bone Joint J.

[30] Fontalis A, Epinette JA, Thaler M, Zagra L, Khanduja V, Haddad FS (2021). Advances and innovations in total hip arthroplasty. SICOT J.

[31] Bergmann G, Graichen F, Rohlmann A (1993). Hip joint loading during walking and running, measured in two patients. J Biomech.

[32] Incavo SJ, Gold JE, Exaltacion JJF, Thompson MT, Noble PC (2011). Does acetabular retroversion affect range of motion after total hip arthroplasty?. Clin Orthop Relat Res.

[33] Tsukeoka T, Tsuneizumi Y, Lee TH (2014). The T-line as an intraoperative landmark for reproducing the native femoral anteversion during hip arthroplasty. Arch Orthop Trauma Surg.

